# Gut Probiotics and Health of Dogs and Cats: Benefits, Applications, and Underlying Mechanisms

**DOI:** 10.3390/microorganisms11102452

**Published:** 2023-09-29

**Authors:** Qing Yang, Zhenlong Wu

**Affiliations:** State Key Laboratory of Animal Nutrition, Department of Companion Animal Science, China Agricultural University, Beijing 100193, China; qing.yang@cau.edu.cn

**Keywords:** probiotics, microbiome, intestinal health, companion animal, dog, cat

## Abstract

Pets (mostly domestic dogs and cats) play an important role in the daily lives of humans and their health has attracted growing attention from pet owners. The intestinal microbiota, a complex microbial community with barrier-protective, nutritional, metabolic, and immunological functions, is integral to host health. Dysbiosis has been related to a variety of diseases in humans and animals. Probiotics have been used in functional foods and dietary supplements to modulate intestinal microbiota and promote host health, which has been introduced in pet dogs and cats in recent years. Various canine- and feline-derived probiotic strains have been isolated and characterized. The administration of probiotics has shown positive effects on the gut health and can alleviate some intestinal diseases and disorders in dogs and cats, although the underlying mechanisms are largely unresolved. In this review, we summarize the current knowledge on the benefits of probiotics and discuss their possible mechanisms in dogs and cats in order to provide new insights for the further development and application of probiotics in pets.

## 1. Introduction

With the advancement of society and improvement in people’s quality of life, an increasing number of households are raising pets or companion animalsthat are dominated by domestic dogs and cats. According to a report by the American Pet Products Association, 70% of American households owned pets by 2021 [1]. Evidence has indicated the benefits of pet ownership on mental and physical health [2,3,4]. It is a global trend that pet owners treat dogs and cats as family members. As dogs and cats have become an important part of human life, their health, especially intestinal health, is attracting increasing attention from pet owners [5].

The microbiota in the gastrointestinal tract (GIT) comprises a complex community of microorganisms, including bacteria, fungi, archaea, protists, and viruses [6]. The GIT microbiota contributes to maintaining intestinal and host homeostasis via multifaceted mechanisms, such as defense against intestinal pathogens, provision of nutrients, facilitation of nutrient digestion and absorption, improvement in barrier function, stimulation of intestinal development, and modulation of the immune system [7,8,9]. Bacterial metabolites, such as short-chain fatty acids (SCFAs), secondary bile acids, and tryptophan metabolites, are key mediators of host−microbiota interactions and play important roles in influencing host health [10,11]. SCFAs are products of bacterial fermentation of dietary fibers, mainly including acetate, propionate, and butyrate, which contribute to intestinal and host health by serving as energy substrates for colonic epithelial cells, maintenance of epithelial barrier integrity, regulation of energy metabolism, and anti-inflammatory effects [12,13]. Secondary bile acids derived from primary bile acids through deconjugation and dihydroxylation by intestinal bacteria possess metabolic and immunomodulatory properties [14,15]. However, it has been demonstrated that dysbiosis or dysfunction of the intestinal microbiota is associated with a variety of diseases, including intestinal disorders and diseases in humans and farm animals [7,16,17]. Perturbation of intestinal microbiota, indicated by decreased microbiota diversity, imbalance of microbiota structure, or shifted metabolite profile, has also been observed in cats and dogs with gut-related disorders, such as chronic enteropathy, inflammatory bowel diseases (IBD), acute uncomplicated diarrhea, and acute hemorrhagic diarrhea syndrome [18,19].

The use of probiotics is a promising approach for manipulating the gut microbiota to promote host health [20,21,22,23]. Studies have shown that probiotics exert prophylactic or therapeutic activities in a variety of diseases such as IBD, antibiotic-associated diarrhea, colon cancer, allergy, type 2 diabetes, and atopic dermatitis in humans [20,21,22,24]. With positive effects such as improving growth performance, promoting intestinal health, and enhancing resistance against infections, probiotics have been explored as promising antibiotic alternatives in animal production [25,26]. An increase in nutrient digestion and absorption, improvement in intestinal morphology, optimization of the gut microbiota and suppressed inflammation have been linked with the health benefits of probiotics in farm animals [23]. Species and strains belonging to *Lactobacillus*, *Bifidobacterium*, *Enterococcus*, and *Saccharomyces* are commonly used probiotics in functional foods and dietary supplements [20,24,27].

Probiotics have been introduced to companion animals in recent years, and current evidence suggests the benefits of probiotics in health promotion and disease prevention in dogs and cats, although related research is not thorough relative to that in humans and livestock species [18,28,29]. Herein, we review the recent advances in the use of probiotics in pet dogs and cats to provide new insights into the further development and application of probiotics in pets.

## 2. The Microbiota in the GIT of Dogs and Cats

### 2.1. The Normal GIT Microbiota of Dogs and Cats

Advancements in high-throughput microbiome techniques such as 16S rRNA gene sequencing, metagenomics, metatranscriptomics, metabolomics, culturomic, and multi-omics make it possible to decipher the composition and function of the microbiota, discover microbiota-associated biomarkers, and reveal host−microbiota interactions [5,6,30,31,32]. An integrated use of culturomic and metagenomic analyses has identified 305 strains of commensal lactic acid bacteria in domestic dogs [33]. Characterization of the normal intestinal microbiota and the identification of microbiota-derived biomarkers associated with GIT diseases are important for understanding host−microbiota interactions and the development of probiotics in cats and dogs.

The canine and feline GIT microbiota remain largely unexplored. Available data suggest that the composition of the GIT microbiota is generally similar between dogs and cats [34,35] (Table 1). Microbial diversity and load increase along the GIT. The numbers of bacteria in the stomach, small intestine, colon, and feces of dogs and cats are generally 10^4^–10^5^ CFU/g, 10^5^–10^9^ CFU/g, 10^9^–10^11^ CFU/g, and 10^8^–10^11^ CFU/g, respectively [36]. In healthy dogs and cats, the stomach microbiota is dominated by *Helicobacter* and *Lactobacillus* [28,36]. Firmicutes, Proteobacteria, Bacteroidetes, Fusobacteria, and Actinobacteria are the major phyla in the intestines of dogs and cats, with variations in abundance in each segment [19,28,36,37]. The fecal microbiota of healthy cats and dogs is dominated by Firmicutes, Proteobacteria, Bacteroidetes, Fusobacteria, and Actinobacteria [18,36]. At the genus level, *Lactobacillus*, *Bifidobacterium*, *Enterococcus*, *Streptococcus,* and *Pediococcus* are dominant in canine feces [38,39]. Moreover, a core microbiota composed of Firmicutes, Bacteroidetes, and Fusobacteria is present in the feces of healthy dogs, among which many bacteria belonging to Clostridia and Bacilli classes are SCFA producers (e.g., *Faecalibacterium* and *Blautia*) or lactic acid bacteria (e.g., *Lactobacillus*) with probiotic properties [18]. *Prevotella* and *Bacteroides* in the phylum Bacteroidetes were abundant in dog feces. *Fusobacterium* is associated with health and is, thus, a potential therapeutic target [18]. Similarly, another study showed that *Fusobacterium*, *Prevotella* 9 (a sub cluster of the *Prevotellaceae* family), and *Bacteroides* are the core intestinal microbiota in both dogs and cats [40]. Many *Bifidobacterium* spp. and lactic acid-producing bacteria have been identified in canine feces [41]. *Lactobacillus* is widespread in the canine and feline intestines and can be the origin of probiotics [27,42].

Despite similarities between the canine and feline intestinal microbiota, differences have been observed between the two species. The diversity of fecal microbiota has been reported to be higher in cats [41,43]. A study showed that the abundances of *Enterococcus*, *Fusobacterium*, and *Megamonas* were higher in the feces of dogs, while multiple genera, including *Bifidobacterium*, *Faecalibacterium*, *Carnobacterium*, *Adlercreutzia*, *Alistipes*, *Collinsella*, *Coprococcus*, *Desulfovibrio*, *Oscillospira*, *Parabacteroides*, *Peptococcus*, *Peptostreptococcus*, *Ruminococcus*, *Slackia*, and *Sutterella*, were more abundant in cats [43]. Another study also identified more abundant and prevalent *Bifidobacterium* in the guts of cats [40]. Dogs and cats have evolved into carnivores and have some anatomical and metabolic characteristics in common, feeding on high-protein diets and having relatively short GITs compared with humans and livestock species [44]. Domestic cats are obligate carnivores that rely on high-protein diets, whereas dogs are metabolically omnivorous and can digest, absorb, and metabolize higher amounts of carbohydrates [44]. The differences in the diet composition could be one of the factors that contribute to shaping divergent gut microbiota between the two species.

### 2.2. Perturbation of Intestinal Microbiota of Dogs and Cats in Intestinal Diseases

An intact intestinal microbiota is essential for maintaining host homeostasis, whereas dysbiosis in the gut microbiota can be linked to a variety of diseases and disorders, such as IBD, diarrhea, obesity, autoimmune diseases, and cancers in humans [7,17]. Current evidence indicates that dysbiosis of the intestinal microbiota is related to various intestinal disorders in dogs and cats, such as chronic intestinal diseases, acute uncomplicated diarrhea, acute hemorrhagic diarrhea syndrome, and bowel cancer [5,35,45,46].

Altered gut microbiota structures and/or metabolite profiles were observed in dogs and cats with intestinal inflammation [35,37,45,46,47]. For example, decreased microbiota diversity, reduction in potentially beneficial bacteria (e.g., *Fusobacterium* and *Faecalibacterium praunitzii*), and overgrowth of *Clostridium perfringens* have been observed in dogs with chronic enteropathy [10,18]. Reductions in *Ruminococcaceae*, *Blautia*, *Faecalibacterium*, and *Turicibacter* have been observed in acute and chronic enteropathy in dogs [48]. Decreased *Bifidobacterium* but increased *Escherichia coli* have been reported in cats with IBD [19]. The disturbances of intestinal microbiota in canine IBD are similar to those in human IBD, characterized by reduced diversity, reduced Firmicutes, increased Proteobacteria, and reduced production of SCFAs and secondary bile acids [37,49]. The identification of microbial signatures associated with health and diseases can promote the development of probiotics. For instance, *Enterococcus hirae*, a dominant species of *Enterococcus* in the small intestinal mucosa of healthy shelter kittens, was found to be negatively associated with enteritis in kittens and was confirmed to have probiotic properties [50,51].

## 3. Mechanisms of Action of Probiotics

Probiotics are live microorganisms with beneficial effects on host health when administered in adequate amounts, as defined by the International Scientific Association for Probiotics and Prebiotics [52]. Probiotics directly target the GIT, but their beneficial effects on the host can be local or systemic [20]. The most commonly studied probiotics include members of lactobacilli (e.g., *Lactobacillus acidophilus*, *Lacticaseibacillus casei*, *Lactobacillus delbrueckii* subsp. *bulgaricus*, *Lacticaseibacillus rhamnosus*, *Lactiplantibacillus plantarum*, and *Limosilactobacillus reuteri*), *Bifidobacterium* (*Bifidobacterium adolescentis*, *Bifidobacterium longum* subsp. *infantis*, *Bifidobacterium breve*, *Bifidobacterium longum*, and *Bifidobacterium bifidum*), and *Saccharomyces* (*Saccharomyces boulardii* and *Saccharomyces cerevisiae*), which have been used as probiotics for a long time with validated safety and efficacy [20,24,27]. Additionally, *Akkermansia muciniphila*, *Faecalibacterium prausnitzii*, *Prevotella copri*, *Bacteroides* spp., *Anaerobutyricum hallii*, *Parabacteroides goldsteinii*, *Roseburia*, and *Propionibacterium* are potentially next-generation probiotics [20,53,54].

Probiotics have been shown to promote health in many diseases, including IBD, diarrhea, colorectal cancer, allergy, type 2 diabetes, and atopic dermatitis, with strain-specific activity [21,24,55]. Probiotics have been proposed to have multiple mechanisms of action. Probiotics confer benefits to host health by interacting with the resident microbiota or by communicating with host cells. Probiotics can improve the balance of the intestinal microbiota, enhance the integrity of the epithelial barrier, and maintain immune homeostasis directly or indirectly [21,37,55,56]. Probiotics inhibit the colonization of pathogens via colonization resistance and production of antimicrobial molecules such as bacteriocins and organic acids (e.g., lactic acid) [20]. Interestingly, strains belonging to the *Lactobacillus* and *Bifidobacterium* genera do not produce butyrate per se, but can cross-feed other commensals to produce butyrate and increase levels of SCFAs, which have multifaceted and important physiological activities [12,57]. Moreover, the beneficial effects of probiotics can be imparted, at least in part, by the induction of host defense peptides (HDPs). Some probiotics, such as *L. casei*, *L. paracasei*, *L. plantarum*, *B. breve*, *A. muciniphila*, *Bacteroides thetaiotaomicron,* and *E. coli Nissle* 1917, stimulate the production of host-derived HDPs in humans and animals [58,59,60,61]. HDPs, also known as antimicrobial peptides, are a group of small cationic amphipathic peptides with a ubiquitous expression in epithelial cells and phagocytes [62]. With antimicrobial and immunomodulatory activities, HDPs constitute an important component of host immune defense [62,63].

The integrity of the intestinal epithelial layer is integral to intestinal homeostasis as the first physical barrier against the external environment and is largely maintained by the mucus layer and tight junctions linking adjacent epithelial cells [8,64,65]. In addition to the inhibition of pathogen colonization and stimulation of HDPs and SCFAs, probiotics contribute to the improvement in epithelial barrier function by promoting the production of mucins and tight junction proteins directly or indirectly [8,20,56,66]. Studies have demonstrated that *Lactobacillus*, *Bifidobacterium*, *A. muciniphila*, and SCFAs stimulate the expression, production, and secretion of mucins [8].

Moreover, probiotics modulate immune functions by stimulating T-cell differentiation, regulating pro- and anti-inflammatory cytokine profiles, and inducing sIgA production [21,55,67]. Furthermore, some probiotic strains, such as *Streptococcus thermophiles* and *Lactobacillus* spp., can produce β-galactosidase and bile salt hydrolase, which are involved in lactose digestion and bile acid metabolism, respectively [20]. Although the mechanisms of action of probiotics are diverse, it is noteworthy that they are heterogeneous and strain-specific [67]. A better understanding of probiotics−host interactions and precise probiotic mechanisms could accelerate the selection of effective probiotics [68].

## 4. Application of Probiotics in Dogs and Cats

Probiotics derived from dogs, cats, and other species have been evaluated in healthy animals and those with intestinal diseases in the form of monostrain or multistrain. The most commonly studied probiotics in dogs and cats are *Lactobacillus*, *Bifidobacterium*, and *Enterococcus* [27,47,69]. Indigenous microbiota is an important source of probiotics owing to the host specificity. Several strains isolated from dogs and cats have exhibited probiotic properties and hold promise for future use [27,28]. The utilization of probiotics in dogs and cats has been shown to improve the gut microbiota balance, modulate inflammation, enhance immune function, and protect against infections caused by enteropathogens [28,70,71,72].

### 4.1. Dogs

Multiple canine-derived bacterial strains with probiotic properties were isolated and identified. A recent study isolated dozens of *Lactobacillus* strains from healthy dogs and revealed their antagonistic activities against *Campylobacter* in vitro [73]. *L. plantarum* strain RW1 isolated from canine feces displayed tolerance to low pH and bile salts, good adhesion to intestinal epithelial cells, and anti-inflammatory activity induced by *Salmonella* infection [74]. *Ligilactobacillus animalis*, identified as the dominant lactic acid bacterium in canine feces, showed various inhibitory effects against enteropathogens [75]. Other studies have isolated bacterial strains from dogs (mainly belonging to the genera *Lactobacillus* and *Enterococcus*) and characterized their probiotic properties in vitro [69,76,77,78,79,80,81,82]. The characteristics of probiotics are associated with host specificity, and it has been proposed that isolation from indigenous microbiota can yield the most optimal strains of probiotics [27,28]. For instance, canine-derived *Enterococcus faecalis* and *Enterococcus faecium* exhibited better adhesion to the intestinal mucosa of dogs than chicken-derived strains of the same species [83]. Similarly, a canine-derived *L. acidophilus* strain showed a higher adhesive ability to canine colonic mucus than strains derived from pigs and humans [80]. Moreover, some studies have confirmed the survivability of potential probiotic strains in gastric acid and the intestines of dogs [84,85].

Studies have indicated that the use of probiotics improves intestinal microbiota composition and changes microbial functions in healthy dogs by adopting 16S rRNA gene sequencing, metagenomics and metabolomics [86,87] (Table 2). Dietary supplementation with *L. reuteri* ZJF036 altered the fecal microbiota composition with increased relative abundances of *Lactobacillus*, but decreased relative abundances of *Turicibacter* and *Blautia* in healthy beagle dogs [88]. Feeding *S. cerevisiae* yeast increased the abundance of *Turicibacter*, decreased the abundance of *E. coli*, increased fecal butyrate, and shifted the microbial functional gene profile in healthy adult beagle dogs [89]. The administration of *Lactiplantibacillus paraplantarum* L-ZS9 changed the intestinal microbial diversity, composition, and metabolism in dogs [90]. Healthy dogs fed the probiotic mixture Slab51^®^ had decreased fecal *C. perfringens* and increased fecal *Bifidobacterium* and *Lactobacillus* [70]. Metabolomics has been used to identify intestinal metabolic changes related to probiotic cheese feeding in healthy beagle dogs [87]. Probiotics also exert immunomodulatory activities in dogs [70,71]. Feeding the probiotic mixture Slab51 elevated fecal IgA and plasma IgG [70]. Multistrain probiotics containing three strains improved the levels of serum IgG and fecal sIgA [71]. However, in some cases, the administration of probiotics did not influence intestinal microbiota or immunological parameters in dogs [91,92].

Moreover, studies have suggested improved fecal quality and reduced nitrogen fermentation through probiotic treatments in dogs [89,93,94]. Feeding *S. cerevisiae* yeast decreased the fecal pH and concentrations of total biogenic amines and ammonia [89]. Dietary supplementation with *Bacillus subtilis* and *Bacillus licheniformis* improved the fecal score, reduced nitrogen fermentation products in feces, and reduced fecal odor in dogs [93]. Dietary supplementation with *Weissella cibaria* JW15 decreased fecal ammonia emission in dogs [94]. In addition, probiotics do not seem to affect the nutrient digestibility or growth performance of dogs [70,88,89,94], with one study indicating that a multistrain probiotic compound improved feed intake and weight gain in dogs [71].

Probiotics confer health benefits in dogs with various intestinal disorders (Table 2). Studies have shown that multistrain probiotics improved clinical remission, suppressed inflammation, and increased epithelial barrier function in dogs diagnosed with IBD [47]. The probiotic mixture Slab51^®^ alleviated the signs of canine colonic dysmotility [95]. A lactic acid bacteria product containing *Limosilactobacillus fermentum*, *L. rhamnosus*, and *L. plantarum* isolated from dogs decreased the frequency of diarrhea in dogs administered with non-steroidal anti-inflammatory drugs [96]. Feeding probiotics containing *Lactobacillus* and *Bifidobacterium* strains increased fecal microbiota diversity, improved microbiota structure, and regulated microbial functional pathways in dogs with diarrhea [86]. Dietary supplementation with *L. rhamnosus* MP01 or *L. plantarum* MP02 isolated from canine milk prevented gastrointestinal infections in puppies [77]. Oral administration of a probiotic mixture accelerated clinical recovery and intestinal microbiota normalization in dogs with acute hemorrhagic diarrhea [97]. A probiotic compound therapy increased the intestinal expression of tight junction proteins in dogs with idiopathic IBD [98].

### 4.2. Cats

*Bifidobacterium* and other lactic acid-producing bacteria, which are largely detected in the feces of healthy cats, are potential sources for the selection of probiotics [41]. A recent study found that strains of *L. plantarum*, *L. rhamnosus*, *L. acidophilus* and *B. adolescentis* isolated from various biotopes of healthy cats showed in vitro probiotic properties such as adhesive activity and antimicrobial activity against causative agents of surgical infection in cats [27]. Another study identified several strains of *L. reuteri*, *L. fermentum*), *E. faecium* and *Pediococcus pentosaceus* from the feces of dogs and cats, although in vivo confirmation of safety and effectiveness is needed for further use [42]. *Bacteroides* sp. CACC 737 isolated from feline feces has potential probiotic properties, as revealed by functional genome analysis [99]. Freeze-dried *E. hirae* isolated from a healthy kitten decreased the diarrhea rate but did not influence the growth or composition of major bacterial phyla [50].

The administration of probiotics promoted the intestinal health of healthy dogs (Table 3). Supplementation with *L. acidophilus* CECT 4529 in the diet improved fecal quality and decreased fecal coliform counts in healthy adult cats [100]. Multistrain probiotics consisting of *S. boulardii* and *Pediococcus acidilactici* increased the production of butyric acid and total SCFAs, reduced concentrations of inflammatory markers myeloperoxidase and calprotectin, and improved the activity of antioxidant enzymes superoxide dismutase and glutathione in the feces of healthy short-haired cats [101]. Interestingly, this study showed that the use of the probiotic mixture did not change fecal microbiota structure in cats [101]. Feeding *E. faecium* SF68 decreased the diarrhea rate in shelter cats but did not affect the diarrhea rate of shelter dogs [102]. Dietary supplementation with *L. acidophilus* DSM13241 increased the numbers of *Lactobacillus* and *L. acidophilus* and decreased the numbers of *Clostridium* spp. and *E. faecalis* in the feces of healthy adult cats [103].

Positive effects of probiotics have also been noted in cats with intestinal disorders (Table 3). Oral administration of *Bacillus licheniformis*-fermented products relieved diarrhea, enriched bacteria belonging to *Clostridium* cluster XIVa, and reduced *C. perfringens* in the feces of cats with chronic diarrhea [104]. A pilot study revealed that the oral administration of a multi-strain probiotic (SLAB51™) composed of eight lactic acid bacteria strains ameliorated clinical signs and increased the abundance of fecal *Lactobacillus* and *Streptococcus* in cats with chronic constipation and idiopathic megacolon [105]. Feeding *E. faecium* strain SF68 improved the fecal quality in cats treated with amoxicillin−clavulanate, which can cause vomiting or diarrhea [106]. 

Although diverse aspects of probiotics have been elucidated, not all activities have been confirmed for every probiotic strain. There are inherent differences between probiotic strains, and the efficacy of probiotics can be influenced by many factors such as species, disease conditions, age, and sex [20,21,71,102,107]. A recent study showed that the bioactivity of probiotics might be sex-specific due to variations in the endocrine and central nervous systems between males and females [107]. 

## 5. Future Directions

Probiotics exert health-promoting effects via direct interaction with the host cells and resident microbiota, or indirectly via microbial metabolites. The intestine hosts the largest and the most complex microbial community. More precise and accurate measurements of microbiota composition and further characterization of microbial functions in the intestine would expand the range and accelerate the selection of probiotics for use in cats and dogs [20]. Although multiple bacterial strains with probiotic characteristics have been isolated from cats and dogs, their health-promotion effects are largely unconfirmed in vivo and there is a shortage of commercial canine/feline-derived probiotics products on the market, which necessitates further and accelerated studies.

It is worth noting that the microbiota can be influenced by many factors, such as species, age, sex, breed, diet, surgery, and antibiotic interventions [17,19,46,47,108,109,110,111]. These factors should be considered when evaluating the in vivo efficacy of probiotics, given that probiotics interact with the gut microbiota. It has been indicated that the administration dose or duration does not impact probiotic health benefits, as illustrated by the application of *L. fermentum* in dogs [72]. Whether this finding applies to other probiotics requires further investigation.

Although several mechanisms of action of probiotics have been proposed, not all have been verified in dogs and cats, warranting more comprehensive measurements and analysis in further research. Moreover, probiotic activities can be heterogeneous and strain-specific; therefore, a precise mechanistic investigation is needed.

Stability is one of the concerns in the application of probiotics. Probiotics such as *Lactobacillus*, *Bifidobacterium*, *A. muciniphila,* and *F. prausnitzii* are sensitive to stressful conditions such as heat, oxygen, pH, enzymes, and bile salts, which compromise their efficacy during processing, storage, distribution, and passage through the GIT [112]. Encapsulation has emerged as an effective strategy to enhance the viability, stability, and efficacy of probiotics [24,112]. Emulsions, gels, powder granules, nanofibers, electrospray capsules, and nano-coatings are commonly used probiotics encapsulation methods [24]. Each approach has specific advantages and disadvantages. However, nano-based encapsulation technology offers higher protection and delivery efficacy for probiotics in contrast with traditional encapsulation methods [113,114]. Furthermore, the single-cell nano-coating encapsulation strategy is considered a more advanced technology than bulk encapsulation methods with nanomaterials such as nanofibers and nanoparticles [113]. Research on encapsulation techniques utilized in probiotics for dogs and cats remains limited. A recent study has indicated that different microencapsulation techniques are differently efficient in maintaining the viability of probiotics during storage and passage through the GIT of cats [115]. Further studies are needed to verify encapsulation techniques for future applications in dogs and cats.

## 6. Conclusions

Maintaining the intestinal health of dogs and cats is becoming increasingly important as pet owners pay more attention to the health of their pets. The utilization of probiotics is an effective strategy to enhance the health of dogs and cats. The administration of probiotics can improve the balance of the intestinal microbiota, suppress inflammation, enhance immune function, and alleviate intestinal disorders in dogs and cats. Various bacterial strains derived from dogs and cats have shown probiotic properties in vitro, but their health benefits need to be confirmed in vivo. Moreover, the mechanisms of action of probiotics need to be further elucidated. Finer characterization of the intestinal microbiota of dogs and cats under different health conditions may facilitate the discovery of novel probiotics for use in pets.

## Figures and Tables

**Table 1:** Distribution of bacterial microbiota along the GIT of dogs and cats.

	Stomach	Small Intestine	Colon	Feces	References
The number of total bacteria	10^4^–10^5^ CFU/g (dogs and cats)	10^5^–10^9^ CFU/g (dogs and cats)	10^9^–10^11^ CFU/g (dogs and cats)	10^8^–10^11^ CFU/g (dogs and cats)	[36]
Dominant bacteria	*Helicobacter* and *Lactobacillus* (dogs and cats)				[28,36]
		Firmicutes Proteobacteria Bacteroidetes Fusobacteria Actinobacteria (dogs and cats)			[19,28,36,37]
				Firmicutes, Proteobacteria, Bacteroidetes, Fusobacteria, and Actinobacteria (dogs and cats); a core microbiota composed of Firmicutes, Bacteroidetes, and Fusobacteria (dogs); *Fusobacterium*, *Prevotella* 9, and *Bacteroides* (dogs and cats)	[18,36,40]

**Table 2:** Effects of probiotics on the intestinal health of dogs.

Probiotics	Main Outcomes	Animals	References
*L. reuteri* ZJF036	fecal *Lactobacillus* ↑ fecal *Turicibacter* and *Blautia* ↓	healthy juvenile beagles; 75 ± 5-day-old; male	[88]
*S. cerevisiae* CNCM I-5660	fecal total biogenic amines and ammonia ↓ fecal pH ↓ fecal butyrate ↑ dysbiosis index ↓ fecal abundance of *Turicibacter* ↑ fecal abundance of *E. coli* ↓	healthy adult beagle dogs; 5-year-old; mixed sex	[89]
probiotic mixture Slab51^®^ containing *S. thermophilus* DSM 32245, *B. lactis* DSM 32246, *B. lactis* DSM 32247, *L. acidophilus* DSM 32241, *L. helveticus* DSM 32242, *L. paracasei* DSM 32243, *L. plantarum* DSM 32244, and *L. brevis* DSM 27961	fecal *C. perfringens* ↓ fecal *Bifidobacterium* and *Lactobacillus* ↑ fecal IgA and plasma IgG ↑	healthy dogs; mixed breeds; 2.5 to 4 years old; mixed sex	[70]
cheese added with *L. reuteri* KACC 92293 and *B. longum* KACC 91563	fecal SCFAs ↑	healthy beagles; mixed sex	[87]
A mixture of *B. subtilis* and *B. licheniformis*	fecal score ↓ fecal odor ↓ fecal biogenic amines ↓	healthy beagle dogs; 4-year-old; mixed sex	[93]
*W. cibaria* JW15	fecal ammonia emission ↓	healthy beagles; 1 to 2 years old; mixed sex	[94]
multistrain probiotics containing *L. casei* Zhang, *L. plantarum* P-8, and *B. animalis* subsp. *lactis* V9	fecal microbial diversity ↑ fecal beneficial bacteria ↑ fecal opportunistic pathogenic bacteria ↓ changes in microbial functional genes	dogs with diarrhea; German Shepherd and Belgium Shepherd dogs; age of 4 months to 13 years; mixed sex	[86]
probiotic mixture Slab51^®^	mast cells in colonic mucosa ↓	dogs with colonic dysmotility; mixed breeds; 2 to 10 years old; mixed sex	[95]
probiotic mixture containing *L. fermentum*, *L. rhamnosus*, and *L. plantarum*	frequency of diarrhea ↓	dogs treated with non-steroidal anti-inflammatory drugs (NSAIDs); mixed breeds; age of 3 months to 14 years; mixed sex	[96]
*L. rhamnosus* MP01, *L. plantarum* MP02	gastrointestinal infections ↓	puppy; German shepherd and Yorkshire; 1-month-old; mixed sex	[77]
probiotic mixture Visbiome^®^ (*L. plantarum* DSM 24730, *S. thermophilus* DSM 24731, *B. breve* DSM 24732, *L. paracasei* DSM 24733, *L. delbrueckii* subsp. *bulgaricus* DSM 24734, *L. acidophilus* DSM 24735, *B. longum* DSM 24736, and *B. infantis* DSM 24737)	clinical recovery ↑ normalization of gut microbiome ↑	dogs with acute hemorrhagic diarrhea; mixed breeds; averagely aged 5.5 or 6 years	[97]
probiotics compound Visbiome^®^	duodenal and colonic expression of E-cadherin, occludin, and zonulin ↑	dogs with idiopathic IBD; mixed pure breeds; mean age of 6.2 and 4.6 years; mixed sex	[98]

The up arrow sign ↑ indicates upregulation while the down arrow sign ↓ indicates downregulation.

**Table 3:** Effects of probiotics on the intestinal health of cats.

Probiotics	Main Outcomes	Animals	References
*L. acidophilus* CECT 4529	fecal quality ↑ fecal Lactobacilli count ↑ fecal Coliform count ↓	healthy adult Maine Coon cats; averagely aged 43.2 or 44.6 months; mixed sex	[100]
a mixture of *S. boulardii* and *P. acidilactici*	fecal butyric acid and total SCFAs ↑ fecal inflammatory markers ↓ fecal antioxidant capacity ↑	healthy short-haired domestic cats; 2 to 4 years old; mixed sex	[101]
*E. hirae* 1002-2	diarrhea rate ↓	healthy kitten; aged < 12 weeks; mixed sex	[50]
*L. acidophilus* DSM13241	fecal *Lactobacillus* ↑ fecal *Clostridium* and *E. faecalis* ↓	healthy domestic shorthair cats; 4 to 5.5 years old	[103]
*B. licheniformis*-fermented product	clinical signs ↓ fecal *Clostridium* cluster XIVa ↑ fecal *C. perfringens* ↓	cats with chronic diarrhea; mixed breeds; age of 3 to 15 years; mixed sex	[104]
multi-strain probiotic (SLAB51™)	clinical symptoms ↓ *Lactobacillus* and *Streptococcus* ↑	cats with chronic constipation and idiopathic megacolon; mixed breeds; 6 to 14 years old; mixed sex	[105]
*E. faecium* strain SF68	fecal quality ↑	cats administered amoxicillin-clavulanate; healthy young adult cats; purpose-bred cat; mixed sex	[106]
*E. faecium* strain SF68	diarrhea rate ↓	shelter cats	[102]

The up arrow sign ↑ indicates upregulation while the down arrow sign ↓ indicates downregulation.

## Data Availability

Not applicable.

## References

[1] 2021–2022 APPA National Pet Owners Survey. https://americanpetproducts.org/Uploads/NPOS/21-22_BusinessandFinance.pdf.

[2] Matchock R.L. (2015). Pet ownership and physical health. Curr. Opin. Psychiatr..

[3] Scoresby K.J., Strand E.B., Ng Z., Brown K.C., Stilz C.R., Strobel K., Barroso C.S., Souza M. (2021). Pet ownership and quality of life: A systematic review of the literature. Vet. Sci..

[4] Tan J.S.Q., Fung W., Tan B.S.W., Low J.Y., Syn N.L., Goh Y.X., Pang J. (2021). Association between pet ownership and physical activity and mental health during the COVID-19 “circuit breaker” in Singapore. One Health.

[5] Alessandri G., Argentini C., Milani C., Turroni F., Cristina Ossiprandi M., van Sinderen D., Ventura M. (2020). Catching a glimpse of the bacterial gut community of companion animals: A canine and feline perspective. Microb. Biotechnol..

[6] Berg G., Rybakova D., Fischer D., Cernava T., Verges M.C., Charles T., Chen X., Cocolin L., Eversole K., Corral G.H. (2020). Microbiome definition re-visited: Old concepts and new challenges. Microbiome.

[7] Hill J.H., Round J.L. (2021). Snapshot: Microbiota effects on host physiology. Cell.

[8] Paone P., Cani P.D. (2020). Mucus barrier, mucins and gut microbiota: The expected slimy partners?. Gut.

[9] de Vos W.M., Tilg H., Van Hul M., Cani P.D. (2022). Gut microbiome and health: Mechanistic insights. Gut.

[10] Gasaly N., de Vos P., Hermoso M.A. (2021). Impact of bacterial metabolites on gut barrier function and host immunity: A focus on bacterial metabolism and its relevance for intestinal inflammation. Front. Immunol..

[11] Lavelle A., Sokol H. (2020). Gut microbiota-derived metabolites as key actors in inflammatory bowel disease. Nat. Rev. Gastroenterol. Hepatol..

[12] Zhang L., Liu C., Jiang Q., Yin Y. (2021). Butyrate in energy metabolism: There is still more to learn. Trends Endocrinol. Metab..

[13] Koh A., De Vadder F., Kovatcheva-Datchary P., Backhed F. (2016). From dietary fiber to host physiology: Short-chain fatty acids as key bacterial metabolites. Cell.

[14] Collins S.L., Stine J.G., Bisanz J.E., Okafor C.D., Patterson A.D. (2023). Bile acids and the gut microbiota: Metabolic interactions and impacts on disease. Nat. Rev. Microbiol..

[15] Cai J., Rimal B., Jiang C., Chiang J.Y.L., Patterson A.D. (2022). Bile acid metabolism and signaling, the microbiota, and metabolic disease. Pharmacol. Ther..

[16] Sun X., Jia Z. (2018). Microbiome modulates intestinal homeostasis against inflammatory diseases. Vet. Immunol. Immunopathol..

[17] Rojo D., Méndez-García C., Raczkowska B.A., Bargiela R., Moya A., Ferrer M., Barbas C. (2017). Exploring the human microbiome from multiple perspectives: Factors altering its composition and function. FEMS Microbiol. Rev..

[18] Pilla R., Suchodolski J.S. (2021). The gut microbiome of dogs and cats, and the influence of diet. Vet. Clin. N. Am. Small Anim. Pract..

[19] Ziese A.L., Suchodolski J.S. (2021). Impact of changes in gastrointestinal microbiota in canine and feline digestive diseases. Vet. Clin. N. Am. Small Anim. Pract..

[20] Sanders M.E., Merenstein D.J., Reid G., Gibson G.R., Rastall R.A. (2019). Probiotics and prebiotics in intestinal health and disease: From biology to the clinic. Nat. Rev. Gastroenterol. Hepatol..

[21] Yan F., Polk D.B. (2020). Probiotics and probiotic-derived functional factors-mechanistic insights into applications for intestinal homeostasis. Front. Immunol..

[22] Li H.Y., Zhou D.D., Gan R.Y., Huang S.Y., Zhao C.N., Shang A., Xu X.Y., Li H.B. (2021). Effects and mechanisms of probiotics, prebiotics, synbiotics, and postbiotics on metabolic diseases targeting gut microbiota: A narrative review. Nutrients.

[23] Ding S., Yan W., Ma Y., Fang J. (2021). The impact of probiotics on gut health via alternation of immune status of monogastric animals. Anim. Nutr..

[24] Gu Q., Yin Y., Yan X., Liu X., Liu F., McClements D.J. (2022). Encapsulation of multiple probiotics, synbiotics, or nutrabiotics for improved health effects: A review. Adv. Colloid Interface Sci..

[25] Lambo M.T., Chang X., Liu D. (2021). The recent trend in the use of multistrain probiotics in livestock production: An overview. Animals.

[26] Su W., Gong T., Jiang Z., Lu Z., Wang Y. (2022). The role of probiotics in alleviating postweaning diarrhea in piglets from the perspective of intestinal barriers. Front. Cell. Infect. Microbiol..

[27] Rudenko P., Vatnikov Y., Sachivkina N., Rudenko A., Kulikov E., Lutsay V., Notina E., Bykova I., Petrov A., Drukovskiy S. (2021). Search for promising strains of probiotic microbiota isolated from different biotopes of healthy cats for use in the control of surgical infections. Pathogens.

[28] Grześkowiak Ł., Endo A., Beasley S., Salminen S. (2015). Microbiota and probiotics in canine and feline welfare. Anaerobe.

[29] Schmitz S., Werling D., Allenspach K. (2015). Effects of ex-vivo and in-vivo treatment with probiotics on the inflammasome in dogs with chronic enteropathy. PLoS ONE.

[30] Nyholm L., Koziol A., Marcos S., Botnen A.B., Aizpurua O., Gopalakrishnan S., Limborg M.T., Gilbert M.T.P., Alberdi A. (2020). Holo-omics: Integrated host-microbiota multi-omics for basic and applied biological research. iScience.

[31] Knight R., Vrbanac A., Taylor B.C., Aksenov A., Callewaert C., Debelius J., Gonzalez A., Kosciolek T., McCall L.I., McDonald D. (2018). Best practices for analysing microbiomes. Nat. Rev. Microbiol..

[32] Suchodolski J.S. (2022). Analysis of the gut microbiome in dogs and cats. Vet. Clin. Pathol..

[33] Kang A.N., Mun D., Ryu S., Jae Lee J., Oh S., Kyu Kim M., Song M., Oh S., Kim Y. (2022). Culturomic-, metagenomic-, and transcriptomic-based characterization of commensal lactic acid bacteria isolated from domestic dogs using caenorhabditis elegans as a model for aging. J. Anim. Sci..

[34] Rodrigues Hoffmann A., Proctor L.M., Surette M.G., Suchodolski J.S. (2016). The microbiome: The trillions of microorganisms that maintain health and cause disease in humans and companion animals. Vet. Pathol..

[35] Hashimoto-Hill S., Alenghat T. (2021). Inflammation-associated microbiota composition across domestic animals. Front. Genet..

[36] Lee D., Goh T.W., Kang M.G., Choi H.J., Yeo S.Y., Yang J., Huh C.S., Kim Y.Y., Kim Y. (2022). Perspectives and advances in probiotics and the gut microbiome in companion animals. J. Anim. Sci. Technol..

[37] Schmitz S., Suchodolski J. (2016). Understanding the canine intestinal microbiota and its modification by pro-, pre- and synbiotics—What is the evidence?. Vet. Med. Sci..

[38] Suchodolski J.S., Camacho J., Steiner J.M. (2008). Analysis of bacterial diversity in the canine duodenum, jejunum, ileum, and colon by comparative 16s rRNA gene analysis. FEMS Microbiol. Ecol..

[39] Kim S.Y., Adachi Y. (2007). Biological and genetic classification of canine intestinal lactic acid bacteria and bifidobacteria. Microbiol. Immunol..

[40] Alessandri G., Milani C., Mancabelli L., Longhi G., Anzalone R., Lugli G.A., Duranti S., Turroni F., Ossiprandi M.C., van Sinderen D. (2020). Deciphering the bifidobacterial populations within the canine and feline gut microbiota. Appl. Environ. Microbiol..

[41] Handl S., Dowd S.E., Garcia-Mazcorro J.F., Steiner J.M., Suchodolski J.S. (2011). Massive parallel 16s rRNA gene pyrosequencing reveals highly diverse fecal bacterial and fungal communities in healthy dogs and cats. FEMS Microbiol. Ecol..

[42] Kim K.T., Kim J.W., Kim S.I., Kim S., Nguyen T.H., Kang C.H. (2021). Antioxidant and anti-inflammatory effect and probiotic properties of lactic acid bacteria isolated from canine and feline feces. Microorganisms.

[43] Jha A.R., Shmalberg J., Tanprasertsuk J., Perry L., Massey D., Honaker R.W. (2020). Characterization of gut microbiomes of household pets in the United States using a direct-to-consumer approach. PLoS ONE.

[44] Deng P., Swanson K.S. (2015). Gut microbiota of humans, dogs and cats: Current knowledge and future opportunities and challenges. Br. J. Nutr..

[45] Rhimi S., Kriaa A., Mariaule V., Saidi A., Drut A., Jablaoui A., Akermi N., Maguin E., Hernandez J., Rhimi M. (2022). The nexus of diet, gut microbiota and inflammatory bowel diseases in dogs. Metabolites.

[46] Huang Z., Pan Z., Yang R., Bi Y., Xiong X. (2020). The canine gastrointestinal microbiota: Early studies and research frontiers. Gut Microbes.

[47] Wernimont S.M., Radosevich J., Jackson M.I., Ephraim E., Badri D.V., MacLeay J.M., Jewell D.E., Suchodolski J.S. (2020). The effects of nutrition on the gastrointestinal microbiome of cats and dogs: Impact on health and disease. Front. Microbiol..

[48] Suchodolski J.S., Markel M.E., Garcia-Mazcorro J.F., Unterer S., Heilmann R.M., Dowd S.E., Kachroo P., Ivanov I., Minamoto Y., Dillman E.M. (2012). The fecal microbiome in dogs with acute diarrhea and idiopathic inflammatory bowel disease. PLoS ONE.

[49] Hernandez J., Rhimi S., Kriaa A., Mariaule V., Boudaya H., Drut A., Jablaoui A., Mkaouar H., Saidi A., Biourge V. (2022). Domestic environment and gut microbiota: Lessons from pet dogs. Microorganisms.

[50] Gookin J.L., Strong S.J., Bruno-Barcena J.M., Stauffer S.H., Williams S., Wassack E., Azcarate-Peril M.A., Estrada M., Seguin A., Balzer J. (2022). Randomized placebo-controlled trial of feline-origin *Enterococcus hirae* probiotic effects on preventative health and fecal microbiota composition of fostered shelter kittens. Front. Vet. Sci..

[51] Ghosh A., Borst L., Stauffer S.H., Suyemoto M., Moisan P., Zurek L., Gookin J.L. (2013). Mortality in kittens is associated with a shift in ileum mucosa-associated enterococci from *Enterococcus hirae* to biofilm-forming *Enterococcus faecalis* and adherent *Escherichia coli*. J. Clin. Microbiol..

[52] Hill C., Guarner F., Reid G., Gibson G.R., Merenstein D.J., Pot B., Morelli L., Canani R.B., Flint H.J., Salminen S. (2014). Expert consensus document. The international scientific association for probiotics and prebiotics consensus statement on the scope and appropriate use of the term probiotic. Nat. Rev. Gastroenterol. Hepatol..

[53] De Filippis F., Esposito A., Ercolini D. (2022). Outlook on next-generation probiotics from the human gut. Cell. Mol. Life Sci..

[54] Chang C.J., Lin T.L., Tsai Y.L., Wu T.R., Lai W.F., Lu C.C., Lai H.C. (2019). Next generation probiotics in disease amelioration. J. Food Drug Anal..

[55] Simon E., Calinoiu L.F., Mitrea L., Vodnar D.C. (2021). Probiotics, prebiotics, and synbiotics: Implications and beneficial effects against irritable bowel syndrome. Nutrients.

[56] Schmitz S.S. (2021). Value of probiotics in canine and feline gastroenterology. Vet. Clin. N. Am. Small Anim. Pract..

[57] Liu H., Wang J., He T., Becker S., Zhang G., Li D., Ma X. (2018). Butyrate: A double-edged sword for health?. Adv. Nutr..

[58] Puértolas-Balint F., Schroeder B.O. (2020). Does an apple a day also keep the microbes away? The interplay between diet, microbiota, and host defense peptides at the intestinal mucosal barrier. Front. Immunol..

[59] Wang J., Zhang W., Wang S., Liu H., Zhang D., Wang Y., Ji H. (2019). Swine-derived probiotic *Lactobacillus plantarum* modulates porcine intestinal endogenous host defense peptide synthesis through TLR2/MAPK/AP-1 signaling pathway. Front. Immunol..

[60] Wu J., Ma N., Johnston L.J., Ma X. (2020). Dietary nutrients mediate intestinal host defense peptide expression. Adv. Nutr..

[61] Pahumunto N., Duangnumsawang Y., Teanpaisan R. (2022). Effects of potential probiotics on the expression of cytokines and human beta-defensins in human gingival epithelial cells and in vivo efficacy in a dog model. Arch. Oral Biol..

[62] Magana M., Pushpanathan M., Santos A.L., Leanse L., Fernandez M., Ioannidis A., Giulianotti M.A., Apidianakis Y., Bradfute S., Ferguson A.L. (2020). The value of antimicrobial peptides in the age of resistance. Lancet Infect. Dis..

[63] Zasloff M. (2002). Antimicrobial peptides of multicellular organisms. Nature.

[64] König J., Wells J., Cani P.D., García-Ródenas C.L., MacDonald T., Mercenier A., Whyte J., Troost F., Brummer R.J. (2016). Human intestinal barrier function in health and disease. Clin. Transl. Gastroenterol..

[65] Grondin J.A., Kwon Y.H., Far P.M., Haq S., Khan W.I. (2020). Mucins in intestinal mucosal defense and inflammation: Learning from clinical and experimental studies. Front. Immunol..

[66] Rose E.C., Odle J., Blikslager A.T., Ziegler A.L. (2021). Probiotics, prebiotics and epithelial tight junctions: A promising approach to modulate intestinal barrier function. Int. J. Mol. Sci..

[67] Plaza-Diaz J., Ruiz-Ojeda F.J., Gil-Campos M., Gil A. (2019). Mechanisms of action of probiotics. Adv. Nutr..

[68] Kleerebezem M., Binda S., Bron P.A., Gross G., Hill C., van Hylckama Vlieg J.E., Lebeer S., Satokari R., Ouwehand A.C. (2019). Understanding mode of action can drive the translational pipeline towards more reliable health benefits for probiotics. Curr. Opin. Biotechnol..

[69] Kubašová I., Lauková A., Hamarová Ľ., Pristaš P., Strompfová V. (2019). Evaluation of enterococci for potential probiotic utilization in dogs. Folia Microbiol. (Praha).

[70] Rossi G., Pengo G., Galosi L., Berardi S., Tambella A.M., Attili A.R., Gavazza A., Cerquetella M., Jergens A.E., Guard B.C. (2020). Effects of the probiotic mixture Slab51^®^ (SivoMixx^®^) as food supplement in healthy dogs: Evaluation of fecal microbiota, clinical parameters and immune function. Front. Vet. Sci..

[71] Xu H., Huang W., Hou Q., Kwok L.Y., Laga W., Wang Y., Ma H., Sun Z., Zhang H. (2019). Oral administration of compound probiotics improved canine feed intake, weight gain, immunity and intestinal microbiota. Front. Immunol..

[72] Strompfová V., Kubašová I., Lauková A. (2017). Health benefits observed after probiotic *Lactobacillus fermentum* CCM 7421 application in dogs. Appl. Microbiol. Biotechnol..

[73] Tomusiak-Plebanek A., Mruk M., Rzaca S., Strus M., Arent Z. (2022). In vitro assessment of anti-*Campylobacter* activity of *lactobacillus* strains isolated from canine rectal swabs. BMC Vet. Res..

[74] Raheem A., Wang M., Zhang J., Liang L., Liang R., Yin Y., Zhu Y., Yang W., Wang L., Lv X. (2022). The probiotic potential of *Lactobacillus plantarum* strain RW1 isolated from canine faeces. J. Appl. Microbiol..

[75] Lee H.J., Lee J.B., Park S.Y., Choi I.S., Lee S.W. (2022). Antimicrobial activity of dominant *Ligilactobacillus animalis* strains in healthy canine feces and their probiotic potential. FEMS Microbiol. Lett..

[76] Jang H.J., Son S., Kim J.A., Jung M.Y., Choi Y.J., Kim D.H., Lee H.K., Shin D., Kim Y. (2021). Characterization and functional test of canine probiotics. Front. Microbiol..

[77] Fernández L., Martínez R., Pérez M., Arroyo R., Rodríguez J.M. (2019). Characterization of *Lactobacillus rhamnosus* MP01 and *Lactobacillus plantarum* MP02 and assessment of their potential for the prevention of gastrointestinal infections in an experimental canine model. Front. Microbiol..

[78] Coman M.M., Verdenelli M.C., Cecchini C., Belà B., Gramenzi A., Orpianesi C., Cresci A., Silvi S. (2019). Probiotic characterization of *Lactobacillus* isolates from canine faeces. J. Appl. Microbiol..

[79] Kumar S., Pattanaik A.K., Sharma S., Jadhav S.E., Dutta N., Kumar A. (2017). Probiotic potential of a *Lactobacillus bacterium* of canine faecal-origin and its impact on select gut health indices and immune response of dogs. Probiotics Antimicrob. Proteins.

[80] Kainulainen V., Tang Y., Spillmann T., Kilpinen S., Reunanen J., Saris P.E., Satokari R. (2015). The canine isolate *Lactobacillus acidophilus* LAB20 adheres to intestinal epithelium and attenuates LPS-induced IL-8 secretion of enterocytes in vitro. BMC Microbiol..

[81] Perelmuter K., Fraga M., Zunino P. (2008). In vitro activity of potential probiotic *Lactobacillus murinus* isolated from the dog. J. Appl. Microbiol..

[82] McCoy S., Gilliland S.E. (2007). Isolation and characterization of *Lactobacillus* species having potential for use as probiotic cultures for dogs. J. Food. Sci..

[83] Hanifeh M., Spillmann T., Huhtinen M., Sclivagnotis Y.S., Gronthal T., Hynonen U. (2021). Ex-vivo adhesion of *Enterococcus faecalis* and *Enterococcus faecium* to the intestinal mucosa of healthy beagles. Animals.

[84] Tang Y., Saris P.E. (2014). Viable intestinal passage of a canine jejunal commensal strain *Lactobacillus acidophilus* LAB20 in dogs. Curr. Microbiol..

[85] Nakamura A., Ohnishi Y., Shirotori K., Matsumoto M. (2015). Evaluation of viability *Bifidobacterium animalis* subsp. Lactis LKM512 in dogs. Benef. Microbes.

[86] Xu H., Zhao F., Hou Q., Huang W., Liu Y., Zhang H., Sun Z. (2019). Metagenomic analysis revealed beneficial effects of probiotics in improving the composition and function of the gut microbiota in dogs with diarrhoea. Food Funct..

[87] Kim Y.J., Park H.E., Lee W.K., Ham J.S., Park S.U., Kim J.G., Im K.H., Kim J.K. (2020). Investigations on metabolic changes in beagle dogs fed probiotic queso blanco cheese and identification of candidate probiotic fecal biomarkers using metabolomics approaches. Metabolites.

[88] Zhao D., Zhang R., Wang J., Zhang X., Liu K., Zhang H., Liu H. (2023). Effect of *Limosilactobacillus reuteri* ZJF036 on growth performance and gut microbiota in juvenile beagle dogs. Curr. Microbiol..

[89] Bastos T.S., Souza C.M.M., Legendre H., Richard N., Pilla R., Suchodolski J.S., de Oliveira S.G., Lesaux A.A., Felix A.P. (2023). Effect of yeast *Saccharomyces cerevisiae* as a probiotic on diet digestibility, fermentative metabolites, and composition and functional potential of the fecal microbiota of dogs submitted to an abrupt dietary change. Microorganisms.

[90] Liu L., Guo S., Chen X., Yang S., Deng X., Tu M., Tao Y., Xiang W., Rao Y. (2021). Metabolic profiles of *Lactobacillus paraplantarum* in biofilm and planktonic states and investigation of its intestinal modulation and immunoregulation in dogs. Food Funct..

[91] Gaspardo A., Zannoni A., Turroni S., Barone M., Sabetti M.C., Zanoni R.G., Forni M., Brigidi P., Pietra M. (2020). Influence of *Lactobacillus kefiri* on intestinal microbiota and fecal IgA content of healthy dogs. Front. Vet. Sci..

[92] Schmitz S., Glanemann B., Garden O.A., Brooks H., Chang Y.M., Werling D., Allenspach K. (2015). A prospective, randomized, blinded, placebo-controlled pilot study on the effect of *Enterococcus faecium* on clinical activity and intestinal gene expression in canine food-responsive chronic enteropathy. J. Vet. Intern. Med..

[93] Bastos T.S., de Lima D.C., Souza C.M.M., Maiorka A., de Oliveira S.G., Bittencourt L.C., Félix A.P. (2020). *Bacillus subtilis* and *Bacillus licheniformis* reduce faecal protein catabolites concentration and odour in dogs. BMC Vet. Res..

[94] Sun H.Y., Kim K.P., Bae C.H., Choi A.J., Paik H.D., Kim I.H. (2019). Evaluation of *Weissella cibaria* JW15 probiotic derived from fermented korean vegetable product supplementation in diet on performance characteristics in adult beagle dog. Animals.

[95] Rossi G., Gioacchini G., Pengo G., Suchodolski J.S., Jergens A.E., Allenspach K., Gavazza A., Scarpona S., Berardi S., Galosi L. (2020). Enterocolic increase of cannabinoid receptor type 1 and type 2 and clinical improvement after probiotic administration in dogs with chronic signs of colonic dysmotility without mucosal inflammatory changes. Neurogastroenterol. Motil..

[96] Herstad K.M.V., Vinje H., Skancke E., Naeverdal T., Corral F., Llarena A.K., Heilmann R.M., Suchodolski J.S., Steiner J.M., Nyquist N.F. (2022). Effects of canine-obtained lactic-acid bacteria on the fecal microbiota and inflammatory markers in dogs receiving non-steroidal anti-inflammatory treatment. Animals.

[97] Ziese A.L., Suchodolski J.S., Hartmann K., Busch K., Anderson A., Sarwar F., Sindern N., Unterer S. (2018). Effect of probiotic treatment on the clinical course, intestinal microbiome, and toxigenic *Clostridium perfringens* in dogs with acute hemorrhagic diarrhea. PLoS ONE.

[98] White R., Atherly T., Guard B., Rossi G., Wang C., Mosher C., Webb C., Hill S., Ackermann M., Sciabarra P. (2017). Randomized, controlled trial evaluating the effect of multi-strain probiotic on the mucosal microbiota in canine idiopathic inflammatory bowel disease. Gut Microbes.

[99] Kim J.A., Jung M.Y., Kim D.H., Kim Y. (2020). Genome analysis of *Bacteroides* sp.CACC 737 isolated from feline for its potential application. J. Anim. Sci. Technol..

[100] Fusi E., Rizzi R., Polli M., Cannas S., Giardini A., Bruni N., Marelli S.P. (2019). Effects of *Lactobacillus acidophilus* D2/CSL (CECT 4529) supplementation on healthy cat performance. Vet. Rec. Open.

[101] Li Y., Ali I., Lei Z., Li Y., Yang M., Yang C., Li L. (2023). Effect of a multistrain probiotic on feline gut health through the fecal microbiota and its metabolite SCFAs. Metabolites.

[102] Bybee S.N., Scorza A.V., Lappin M.R. (2011). Effect of the probiotic *Enterococcus faecium* SF68 on presence of diarrhea in cats and dogs housed in an animal shelter. J. Vet. Intern. Med..

[103] Marshall-Jones Z.V., Baillon M.L., Croft J.M., Butterwick R.F. (2006). Effects of *Lactobacillus acidophilus* DSM13241 as a probiotic in healthy adult cats. Am. J. Vet. Res..

[104] Lee T.W., Chao T.Y., Chang H.W., Cheng Y.H., Wu C.H., Chang Y.C. (2022). The effects of *Bacillus licheniformis*-fermented products on the microbiota and clinical presentation of cats with chronic diarrhea. Animals.

[105] Rossi G., Jergens A., Cerquetella M., Berardi S., Di Cicco E., Bassotti G., Pengo G., Suchodolski J.S. (2018). Effects of a probiotic (SLAB51™) on clinical and histologic variables and microbiota of cats with chronic constipation/megacolon: A pilot study. Benef. Microbes.

[106] Torres-Henderson C., Summers S., Suchodolski J., Lappin M.R. (2017). Effect of *Enterococcus faecium* strain SF68 on gastrointestinal signs and fecal microbiome in cats administered amoxicillin-clavulanate. Top. Companion Anim. Med..

[107] Snigdha S., Ha K., Tsai P., Dinan T.G., Bartos J.D., Shahid M. (2022). Probiotics: Potential novel therapeutics for microbiota-gut-brain axis dysfunction across gender and lifespan. Pharmacol. Ther..

[108] You I., Kim M.J. (2021). Comparison of gut microbiota of 96 healthy dogs by individual traits: Breed, age, and body condition score. Animals.

[109] Garrigues Q., Apper E., Chastant S., Mila H. (2022). Gut microbiota development in the growing dog: A dynamic process influenced by maternal, environmental and host factors. Front. Vet. Sci..

[110] Whittemore J.C., Stokes J.E., Price J.M., Suchodolski J.S. (2019). Effects of a synbiotic on the fecal microbiome and metabolomic profiles of healthy research cats administered clindamycin: A randomized, controlled trial. Gut Microbes.

[111] Rochegue T., Haenni M., Mondot S., Astruc C., Cazeau G., Ferry T., Madec J.Y., Lupo A. (2021). Impact of antibiotic therapies on resistance genes dynamic and composition of the animal gut microbiota. Animals.

[112] Yao M., Xie J., Du H., McClements D.J., Xiao H., Li L. (2020). Progress in microencapsulation of probiotics: A review. Compr. Rev. Food Sci. Food Saf..

[113] Centurion F., Basit A.W., Liu J., Gaisford S., Rahim M.A., Kalantar-Zadeh K. (2021). Nanoencapsulation for probiotic delivery. ACS Nano.

[114] Xu C., Ban Q., Wang W., Hou J., Jiang Z. (2022). Novel nano-encapsulated probiotic agents: Encapsulate materials, delivery, and encapsulation systems. J. Control. Release.

[115] Rodrigues B.M., Olivo P.M., Osmari M.P., Vasconcellos R.S., Ribeiro L.B., Bankuti F.I., Pozza M.S.S. (2020). Microencapsulation of probiotic strains by lyophilization is efficient in maintaining the viability of microorganisms and modulation of fecal microbiota in cats. Int. J. Microbiol..

